# Copper-Induced Neurodegenerative Disorders and Therapeutic Potential of Curcumin-Loaded Nanoemulsion

**DOI:** 10.3390/toxics13020108

**Published:** 2025-01-29

**Authors:** Govind Hake, Akshada Mhaske, Rahul Shukla, Swaran Jeet Singh Flora

**Affiliations:** 1 Department of Pharmaceutics, National Institute of Pharmaceutical Education and Research-Raebareli, Near CRPF Base Camp, Bijnor-Sisendi Road, Sarojini Nagar, Lucknow 226002, India; govindkh.765mspe21@niperrbl.ac.in (G.H.);; 2 Era College of Pharmacy, Era Lucknow Medical University, Sarfarajgunj, Lucknow Hardoi Road, Lucknow 226002, India

**Keywords:** copper toxicity, Wilson’s disease, mitochondrial oxidative stress, chelation therapy, neurotoxicity, herbal molecules

## Abstract

Copper accumulation in neurons induces oxidative stress, disrupts mitochondrial activity, and accelerates neuronal death, which is central to the pathophysiology of neurodegenerative diseases like Wilson disease. Standard treatments for copper toxicity, such as D-penicillamine, trientine, and chloroquine, are frequently associated with severe side effects, creating a need for safer therapeutic alternatives. To address this, we developed a curcumin-loaded nanoemulsion (CUR-NE) using the spontaneous emulsification technique, aimed at enhancing the bioavailability and therapeutic efficacy of curcumin. The optimized nanoemulsion displayed a particle size of 76.42 nm, a zeta potential of −20.4 mV, and a high encapsulation efficiency of 93.69%, with a stable and uniform structure. The in vitro tests on SH-SY5Y neuroblastoma cells demonstrated that CUR-NE effectively protected against copper-induced toxicity, promoting significant cellular uptake. Pharmacokinetic studies revealed that CUR-NE exhibited a longer half-life and extended circulation time compared to free curcumin. Additionally, pharmacodynamic evaluations, including biochemical assays and histopathological analysis, confirmed that CUR-NE provided superior neuroprotection in copper overload conditions. These results emphasize the ability of CUR-NE to augment the therapeutic effects of curcumin, presenting a novel approach for managing copper-induced neurodegeneration. The study highlights the effectiveness of nanoemulsion-based delivery platforms in improving chelation treatments for neurological diseases.

## 1. Introduction

Copper (Cu) toxicity occurs when excess copper accumulates in the body due to impaired regulation, especially in the liver and brain. While Cu is essential for biological functions, excessive amounts lead to toxicity. This is often caused by defects in Cu-transporting proteins like ATP7A or ATP7B [1,2]. In the brain, excess Cu generates reactive oxygen species (ROS), leading to oxidative damage to lipids, proteins, and DNA, which damages neurons. Cu also disrupts mitochondrial function, reducing energy production and exacerbating oxidative stress, particularly in regions like the basal ganglia, impairing motor control and cognition [3]. Cu accumulation activates glial cells, triggering neuroinflammation, which worsens neuronal damage. The combined effects of oxidative stress, mitochondrial dysfunction, and inflammation impair cellular repair, contributing to neurodegeneration [4]. First-line treatment for Cu toxicity typically involves the use of metal chelation therapy and zinc supplementation. Metal chelators such as D-penicillamine, dimercaptosuccinic acid, and trientine are used to bind excess Cu, facilitating its excretion through urine. Concurrently, zinc salts are administered to reduce Cu absorption in the gastrointestinal tract, limiting its systemic circulation [5,6]. These combined strategies effectively manage Cu overload and prevent further toxic effects on organs such as the liver and brain [7]. Synthetic chelators used to treat Cu toxicity can lead to several adverse effects, such as kidney damage, gastrointestinal discomfort, and bone marrow suppression. Additionally, they necessitate careful monitoring due to potential Cu deficiency and interactions with other treatments. Zinc therapy, although beneficial, may be less effective in severe cases and can cause side effects with prolonged use [8,9]. Herbal chelators present a promising alternative, offering a safer profile with fewer side effects. These natural agents may help modulate Cu levels and combat oxidative damage, providing a viable long-term treatment option with a reduced risk of adverse effects [10]. Curcumin (CUR) is a natural bioactive polyphenol obtained by the extraction process from rhizomes of *Curcuma longa* L. [11]. Numerous studies have reported that CUR exhibits antioxidant, anticancer, anti-inflammatory, antimicrobial, antiepileptic, antidepressant, immunomodulatory, neuroprotective, antiapoptotic, and antiproliferative properties [12]. It acts as an ROS scavenger, increasing the glutathione (GSH) level by inducing the glutathione cysteine ligase. CUR may protect the brain from Aβ toxicity in Alzheimer’s disease animal models by chelating copper sulfate (Cu^2+^) ions [13]. With its various applications, the most common problems concerning the biopharmaceuticals of CUR are poor aqueous solubility, instability, rapid metabolism by phase II reaction in the hepatocytes with biliary excretion, and poor intestinal permeability, so it is challenging to incorporate into aqueous formulations [14].

The clinical applications of CUR are diverse, with promising potential in various therapeutic areas. In cancer treatment, CUR’s ability to bind Cu^2+^ has been linked to its effectiveness in limiting tumor growth and preventing angiogenesis [15]. It reduces inflammation by chelating metals and suppressing pathways like NF-κB. It also boosts antioxidant defenses by activating key enzymes such as catalase and superoxide dismutase [16]. Furthermore, CUR has shown applicability in neutralizing the harmful effects of heavy metals like lead, mercury, and cadmium by mitigating oxidative damage. However, its broader clinical use faces obstacles, such as limited absorption in the body, non-selective metal-binding that may affect essential minerals like iron and zinc, and the need for higher doses that can lead to gastrointestinal discomfort [17]. Additionally, CUR’s interactions with certain medications, particularly those involved in blood clotting or acid regulation, require careful consideration. Although early-stage research provides encouraging results, more comprehensive clinical studies are needed to validate its therapeutic potential, underscoring the importance of developing methods to enhance its absorption and overall efficacy [18]. To address the physicochemical challenges associated with CUR, lipid-based encapsulation technologies, such as nanoemulsion, could be explored. Nanoemulsion can efficiently solubilize large amounts of lipophilic compounds within oil droplets, which are stabilized by a surfactant film at the interface [19]. In this study, we focused on developing a stable CUR-loaded nanoemulsion and assessed its effectiveness in counteracting Cu^2+^-induced neurotoxicity in a rodent model [20,21]. CUR was encapsulated in nanoemulsion to enhance its solubility, stability, and bioavailability, overcoming its poor water solubility and rapid metabolism. The nano-sized droplets improve absorption, cellular penetration, and targeted delivery, boosting its efficacy for clinical use. Ginger oil nanoemulsion, rich in antioxidant compounds like 6-shogaol and gingerol, combat metal toxicity by reducing oxidative stress. The nanoemulsion technology enhances bioavailability, stability, and targeted delivery, offering a promising natural solution for managing metal-induced damage.

## 2. Result and Discussion

### 2.1. Pre-Formulation Analysis

#### 2.1.1. Solubility Evaluation of Curcumin

The solubility of CUR in various components of the formulation, including the oil phase, surfactants, and cosurfactants, was assessed. The analysis was performed using ginger oil as the primary oil phase, along with diethylene glycol monoethyl ether (DGME), Tween 80, ethanol, PEG 400, and Cremophor EL. The solubility values observed for CUR in these components were 1.47 ± 0.91 mg/mL in ginger oil, 15.3 ± 1.68 mg/mL in DGME, 8.95 ± 0.81 mg/mL in Tween 80, 5.29 ± 1.74 mg/mL in ethanol, 20.0 ± 1.55 mg/mL in PEG 400, and 4.13 ± 1.21 mg/mL in Cremophor EL [22]. The solubility profile is depicted in Figure S1A,B.

#### 2.1.2. Selection of Oil, Surfactant, and Co-Surfactant

The oil selection was based on solubility and miscibility of CUR in oil. Ginger oil was preferred based on its solubility with other excipients and antioxidant activity in various neurodegenerative disorders [23]. Tween 80 and polyethylene glycol 400 (PEG 400) surfactants were selected in preliminary studies. Transcutol P and ethanol (EoH) were selected as a cosurfactant due to the property of permeation enhancement [24]. It was noted that ginger oil was miscible in Tween 40, 60, and 80; Transcutol P; Cremophor EL; and ethanol, and it was immiscible with a span of 20, 80, and PEG 400. Based on the evaluation, the above-mentioned surfactants and cosurfactants were selected for preliminary formulation batches.

#### 2.1.3. Design of Pseudo-Ternary Phase Diagram

Pseudo-ternary phase diagrams were developed using ginger oil as the oil phase, with surfactant mixture of Tween 80 combined with ethanol, PEG 400 and DGME as cosurfactants, and water as the aqueous phase. Nanoemulsion prepared with a S_mix_ of PEG 400 and ethanol exhibited a biphasic appearance. Adjusting the PEG 400-to-ethanol did not significantly expand the clear nanoemulsion region. Increasing the ginger oil concentration from 5% to 15% (*w*/*w*) substantially reduced the transparent nanoemulsion zone, indicating a concentration-dependent effect. Increasing surfactant levels, along with PEG 400 and ethanol, led to a larger clear nanoemulsion region. Excessive surfactant concentrations have been associated with safety concerns due to potential toxicity. Among the tested formulations, the combination of Tween 80 and ethanol produced the clearest nanoemulsion, as depicted in Figure S2. In contrast, increasing the ethanol ratio to 1:2 reduced the nanoemulsion region, likely due to a decrease in the interfacial barrier between the oil and aqueous phases. Varying the Tween 80 ratio showed no significant changes in the clear region, which could be attributed to the inherent phase behaviour of the ternary system. Nanoemulsion formulated with Tween 80 and DGME showed slightly smaller clear regions compared to the Tween 80 and ethanol system. Nanoemulsions are kinetically stable systems, formed at precise concentrations of oil, surfactant, cosurfactant, and water, and exhibit no phase separation, creaming, or cracking. Formulations identified from the phase diagrams underwent stress stability testing, including centrifugation, freeze–thaw cycles, and heating–cooling cycles. Additionally, temperature fluctuations during testing disrupt stability by causing phase separation and altering droplet distribution due to curvature free energy changes. Combinations that showed no evidence of phase separation, creaming, cracking, coalescence, or phase inversion during stress stability tests were deemed stable and selected for further studies, as summarized in Table 1.

### 2.2. Characterization of CUR-NE Formulation

**Figure 1:**
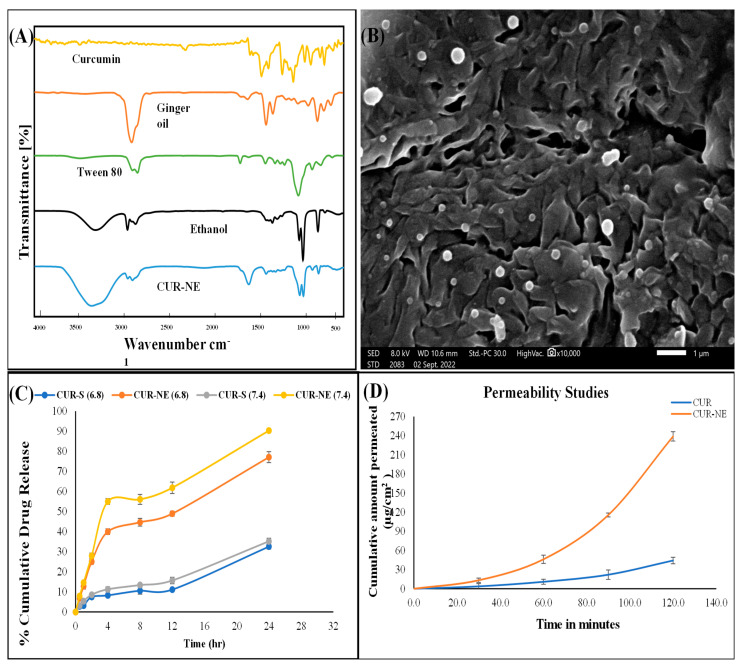
(**A**) FTIR spectra of curcumin, ginger oil, Tween 80, ethanol, and CUR-NE. (**B**) SEM micrograph of CUR-NE showing spherical, small, rounded particles. (**C**) Cumulative drug release (%) from CUR suspension and CUR-NE in phosphate buffer at pH 6.8 and 7.4, with all samples evaluated in triplicate. (**D**) The permeability of the prepared nanoformulation was assessed using the intestinal sac model, with all samples analysed in triplicate. 1: 1 is part of unit of wavenumber. It should read as cm^−1^.

The particle size of CUR-loaded nanoemulsion (CUR-NE) plays a crucial role in optimizing its oral bioavailability, as smaller particles can enhance drug absorption and improve permeation across the intestinal barrier. The particle size, polydispersity index, and zeta potential of the CUR-NE were measured using a Zetasizer. The optimized formulations results showed a hydrodynamic diameter of 76.42 nm with a PDI of 0.266, indicating a narrow and uniform particle size distribution. The zeta potential, which provides insight into the stability of the formulation, was at −20.34 mV, suggesting that the CUR-NE is stable. This negative zeta potential is likely due to the anionic nature of CUR. To further examine the particle morphology, scanning electron microscopy (SEM) was used, revealing that the particles were spherical, evenly spaced, and mostly smaller than 100 nm. The particle size obtained from the Zetasizer was notably smaller than that observed in the SEM analysis [19]. The percentage drug content in the nanocarrier was found to be 93.69%, indicating efficient encapsulation of CUR within the oil droplets. FTIR analysis showed distinct functional group peaks for each excipient. In the CUR-NE, the intensity of CUR-specific peaks was reduced, likely due to its encapsulation in the internal phase of the nanoemulsion. Figure 1A displays the FTIR peaks for CUR, excipients, and the formulation. Ketone stretching (-C=O) was observed at 1542.65 cm^−1^ in CUR and at 1645.26 cm^−1^ in CUR-loaded nanoemulsion. Aromatic -C-H bending appeared at 883.44 cm^−1^ in ginger oil and at 878.62 cm^−1^ in CUR-loaded nanoemulsion. The alcoholic -O-H bending was observed at 1044.99 cm^−1^ in ethanol and at 1045.24 cm^−1^ in the nanoemulsion. The ether -C-O stretching was seen at 1095.36 cm^−1^ in Tween 80 and at 1085.54 cm^−1^ in CUR-loaded nanoemulsion. These observations confirm that all characteristic peaks of the excipients were present in the CUR-NE. The FTIR spectra of CUR, ginger oil, Tween 80, ethanol, and the CUR-loaded nanoemulsion are shown in Figure 1A. The SEM images revealed that the CUR-NE particles were spherical, with a smooth surface morphology. Minimal aggregation of the particles was observed, which can be attributed to the surfactant concentration used in the formulation (Figure 1B).

### 2.3. In Vitro Release Profile Analysis

The in vitro drug release behaviour of the CUR solution and optimized nanoemulsion was assessed using the dialysis bag method across different physiological pH levels (6.8, and 7.4). After 2 h, the cumulative CUR release was 7.46%, and 8.56% for the drug solution, while for the CUR -loaded nanocarriers, it was 25.00% and 27.99%, respectively, in (6.8 and 7.4) buffer. These findings demonstrate that CUR release from the nanocarriers was notably faster than from the drug solution. The release data are depicted in Figure 1C. This increased release rate can be attributed to the presence of surfactants and cosurfactants in the formulation, which reduce interfacial barriers and facilitate drug diffusion into the release medium. Release kinetics analysis further confirmed that the CUR release from the nanocarriers followed a first-order kinetic model [25].

### 2.4. Ex Vivo Intestinal Permeability Studies

The ex vivo intestinal permeation of free CUR and CUR-NE was evaluated using the non-everted intestinal gut sac technique. At 2 h, the cumulative permeation of CUR per unit area was recorded, with free CUR reaching 44.26 µg/cm^2^, while CUR-NE demonstrated a significantly higher permeation of 238.96 µg/cm^2^. These results indicate that CUR-NE exhibited a significantly higher absorption compared to free CUR at various time intervals. The cumulative permeation of CUR over time is illustrated in Figure 1D.

### 2.5. Thermodynamic Stability Studies

The physicochemical stability of the prepared nano-formulation was evaluated by monitoring changes in particle size, polydispersity index (PDI), and zeta potential over a one-month period at 4 °C, 25 °C, and 45 °C. The results showed that the CUR-NE formulation remained stable under different storage temperature conditions. The stability outcome is shown in Table 2.

### 2.6. Cell Line Study

#### 2.6.1. Cell Viability Analysis

**Figure 2:**
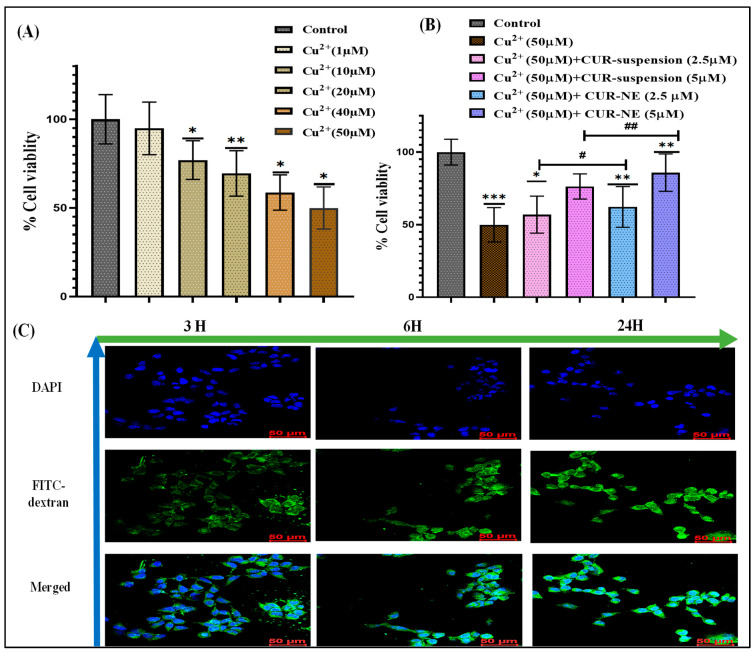
(**A**) Cell viability analysis of Cu^2+^ at concentrations ranging from 1 to 50 µM. (**B**) Cell viability analysis of Cu^2+^ (50 µM) with various treatment groups. (**C**) Confocal microscopy images illustrating the cellular uptake of FITC-loaded nanoemulsion in SH-SY5Y cells at 3, 6, and 24 h incubation time points (40× magnification). Data were analyzed using one-way ANOVA with Dunnett’s multiple comparison. All experiments were conducted in triplicates (*n* = 3). Data were analyzed using one-way ANOVA with Dunnett’s multiple comparison. All treatment groups were statistically compared to the control group to assess differences in the measured outcomes (denoted with *, *** 0.0001 < *p* ≤ 0.001, ** 0.001 < *p* ≤ 0.01, * *p* ≤ 0.05). The CUR vs. CUR-NE groups were statistically analyzed using a one-way ANOVA followed by the Sidak multiple comparisons test (denoted with #). ##: 0.001 < *p* ≤ 0.01, #: *p* ≤ 0.05.

The SH-SY5Y cells were exposed to varying concentrations of Cu^2+^ (ranging from 1 to 50 μM) to identify the most suitable dose for subsequent experiments. Cu^2+^ treatment resulted in a concentration-dependent decrease in cell viability, with a 50 μM concentration showing a significant reduction of 49.98%. Based on these results, 50 μM Cu^2+^ was chosen to induce neurotoxicity in further studies. Additional tests revealed that treatment with equivalent CUR suspension and CUR-NE, at concentrations of 2.5 μM and 5 μM, effectively mitigated the Cu^2+^-induced toxicity and enhanced cell viability. The cell viability analysis is represented in Figure 2A,B.

#### 2.6.2. Cellular Uptake Evaluation

The uptake of the FITC-labeled formulation in SH-SY5Y cells was monitored at 3, 6, and 24 h. The analysis showed a gradual, time-dependent increase, with the highest uptake occurring at 24 h, a moderate level at 6 h, and the lowest at 3 h. This pattern indicates progressive accumulation of the FITC-labeled nanoformulation within the cells as the incubation time increased. The uptake dynamics are presented in Figure 2C.

### 2.7. In Vivo Evaluation

#### 2.7.1. Pharmacokinetic Assessment

The investigation aimed to compare the pharmacokinetics and tissue distribution of CUR-NE with CUR suspension following oral administration. Samples from plasma, brain, liver, and kidneys were collected to evaluate the absorption, distribution, and elimination profiles of both regimens. CUR-NE showed a significantly higher brain concentration, reaching 1229.64 ± 88.99 ng/g, compared to the suspension. In plasma, CUR-NE achieved a peak concentration of 1207.35 ± 79.14 ng/mL, while the suspension displayed a higher value of 1507.33 ± 84.11 ng/mL. Liver concentrations for the suspension peaked at 1392.163 ± 91.12 ng/g, whereas CUR-NE reached a maximum of 921.04 ± 75.33 ng/g. Similarly, kidney concentrations were highest for the suspension at 1391.53 ± 52.99 ng/g, whereas CUR-NE reached 736.53 ± 61.69 ng/g.

**Figure 3:**
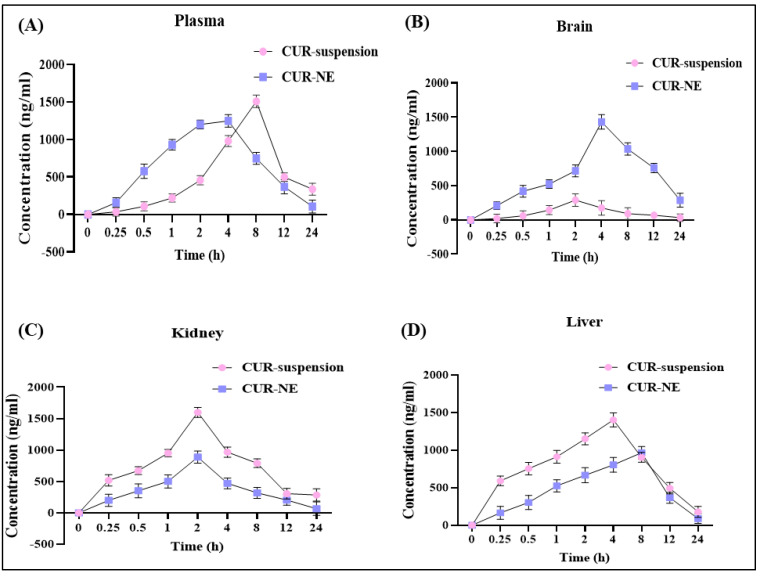
(**A**) Pharmacokinetic estimation of CUR in plasma at various time points. (**B**) Pharmacokinetic estimation of CUR in the brain at different time points. (**C**) Pharmacokinetic estimation of CUR in the kidney at multiple time points. (**D**) Pharmacokinetic estimation of CUR in the liver at several time points. All experiments were conducted with a sample size of *n* = 6.

The AUC_Brain_ for CUR-NE was significantly higher 16,330.34 ng/mg·h, in contrast to 2719.98 ng/g·h for the suspension, suggesting a marked improvement in bioavailability. In terms of brain concentration, CUR-NE achieved a peak value at (T_max_) of 4 h, while the suspension reached only 9 h, indicating superior brain targeting. Furthermore, the brain targeting index for CUR-NE was 4.7, underscoring its enhanced ability to effectively accumulate in brain tissues. These findings are further detailed in Figure 3A–D and Table 3.

#### 2.7.2. Pharmacodynamic Evaluation

**Figure 4:**
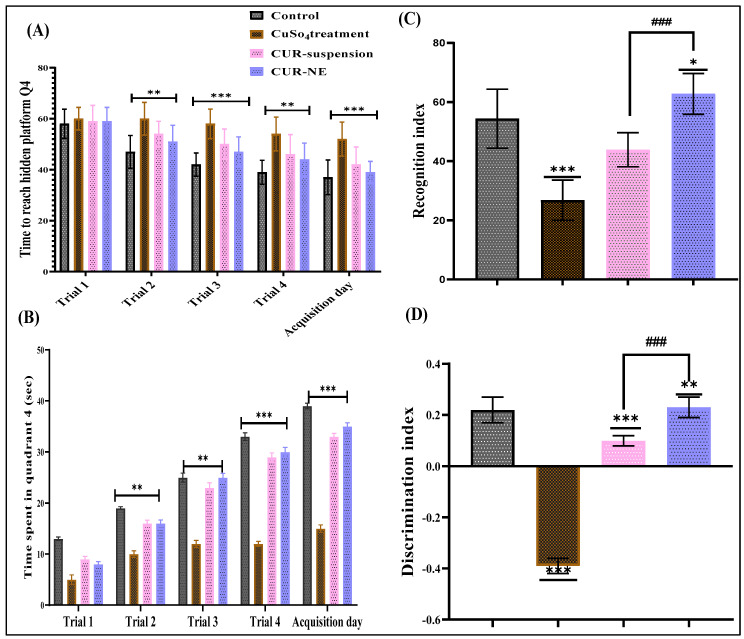
(**A**) Morris’s water maze (MWM) test showing total time to reach the platform in Q4 for control, disease control, Cu+ CUR, and Cu+ CUR-NE groups (*n* = 5). (**B**) Graphical representation of the time spent in Q4 (*n* = 5). (**C**) Recognition index for different treatment groups. (**D**) Discrimination index for different treatment groups. Statistical analysis for panels (**A**,**B**) was performed using two-way ANOVA with Dunnett’s multiple comparison; all groups were compared with control. All experiments were conducted with a sample size of *n* = 6. For (**C**,**D**) graph, data were analyzed using one-way ANOVA with Dunnett’s multiple comparison. All treatment groups were statistically compared to the control group to assess differences in the measured outcomes (denoted with *, *** 0.0001 < *p* ≤ 0.001, ** 0.001 < *p* ≤ 0.01, * *p* ≤ 0.05). The CUR vs. CUR-NE groups were statistically analyzed using a one-way ANOVA followed by the Sidak multiple comparisons test (denoted with ###); ### 0.0001 < *p* ≤ 0.001.

The Morris water maze (MWM) test was conducted to compare the effects of orally administered CUR-NE with those of CUR suspension. The parameters assessed included the time taken to locate the platform and the duration spent in the target zone. These results were analysed across the control group, Cu^2+^-treated group, and Cu^2+^-treated groups with treatments groups. The study showed that rats receiving CUR-NE exhibited memory retention comparable to the control group, whereas Cu^2+^-treated rats demonstrated impaired memory. While rats treated with CUR suspension also showed improved memory, the effect was less significant compared to those receiving CUR-NE (Figure 4A,B).

Results from the Novel Object Recognition Test (NORT) are presented in Figure 4C,D. During the probe test (R2), rats exposed to Cu^2+^ displayed a marked decrease in preference for the novel object compared to the control group. Conversely, rats treated with Cu^2+^ + CUR and Cu^2+^ + CUR-NE showed significant improvements in novel object preference compared to the Cu^2+^ group. In the treatment groups (CUR and CUR-NE during T2), a strong preference for the novel object was observed, indicating notable cognitive improvement compared to the Cu^2+^-treated group.

#### 2.7.3. Biochemical Estimation of Neuronal Oxidative Stress Markers

**Figure 5:**
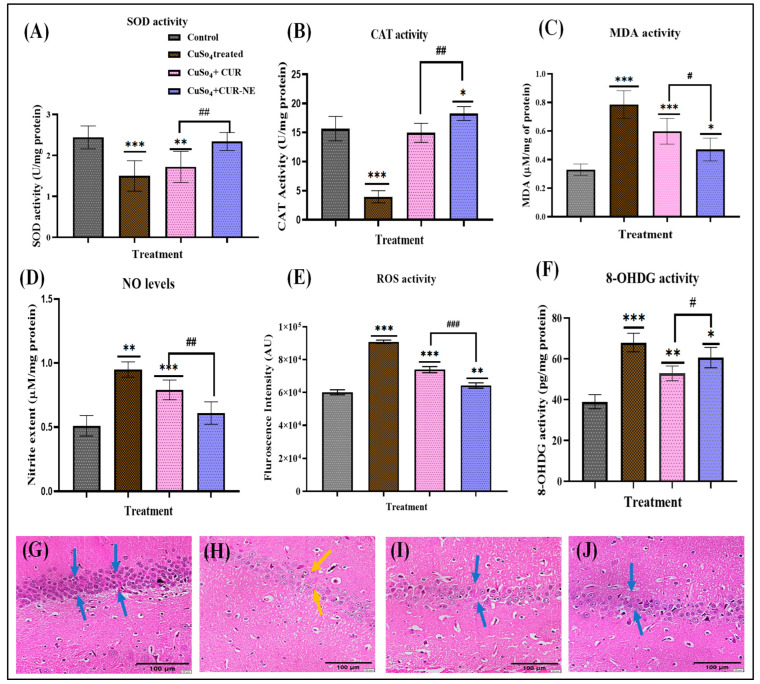
(**A**) Evaluation of superoxide dismutase (SOD) activity. (**B**) Evaluation of catalase (CAT) activity. (**C**) Measurement of malondialdehyde (MDA) levels. (**D**) Assessment of nitric oxide (NO) activity. (**E**) Determination of reactive oxygen species (ROS) levels. (**F**) Evaluation of 8-hydroxy-2′-deoxyguanosine (8-OHdG) levels. (**G**) Histopathological analysis of brain tissue in the control group. (**H**) Histopathological analysis of brain tissue in the Cu^2+^ control group. (**I**) Histopathological evaluation of brain tissue in the Cu^2+^ + CUR suspension group. (**J**) Histopathological analysis of brain tissue in the Cu^2+^ + CUR-NE group. All experiments were conducted with a sample size of *n* = 6. Data were analyzed using one-way ANOVA with Dunnett’s multiple comparison. All treatment groups were statistically compared to the control group to assess differences in the measured outcomes (denoted with *, *** 0.0001 < *p* ≤ 0.001, ** 0.001 < *p* ≤ 0.01, * *p* ≤ 0.05). The CUR vs. CUR-NE groups were statistically analyzed using a one-way ANOVA followed by the Sidak multiple comparisons test (denoted with #); ###: 0.0001 < *p* ≤ 0.001, ##: 0.001 < *p* ≤ 0.01, #: *p* ≤ 0.05.

Superoxide dismutase (SOD) is crucial for the scavenging of reactive oxygen species (ROS). The highest SOD activity was observed in the control group, followed by the Cu^2+^ + CUR-NE and Cu^2+^ + CUR treatment groups. The lowest SOD activity was found in the group exposed to Cu^2+^ alone [26]. The graphical representation of SOD estimation is represented in Figure 5A.

Catalase (CAT) activity changes serve as an indicator of oxidative stress levels. The highest catalase activity was observed in the control group, followed by the Cu^2+^ + CUR-NE and Cu^2+^ + CUR groups. In contrast, the group exposed to Cu^2+^ alone showed the lowest catalase activity. The catalase activity data are illustrated in Figure 5B. In the malondialdehyde (MDA) assessment, treatment with CUR-NE and CUR reduced MDA levels compared to the Cu^2+^ group, as shown in Figure 5C. Nitrite (NO) levels, which indicate the production of nitrogen species that can damage cellular structures, were elevated in the Cu^2+^-induced neurotoxicity group compared to the other groups. Figure 5D shows that treatment with CUR and CUR-NE effectively reduced excessive nitrite levels, offering potential protection against oxidative stress caused by Cu^2+^ toxicity. In neurodegenerative diseases, oxidative stress results from ROS-induced damage to neurons, potentially leading to their degeneration and dysfunction. The elevated ROS levels in Cu^2+^-induced rats indicate a significant increase in oxidative stress, highlighting the neurotoxic effects of Cu^2+^ [27]. The Cu^2+^ + CUR and Cu^2+^ + CUR-NE groups exhibited a decrease in ROS activity, indicating that CUR and CUR-NE may promote the chelation of Cu^2+^. The ROS measurement results are depicted in Figure 5E. The molecule 8-hydroxy-2′-deoxyguanosine (8-OHdG) is a prominent marker of oxidative stress, specifically indicating DNA damage. In the case of in vivo Cu^2+^ -induced neurotoxicity, higher levels of 8-OHdG signify an increase in oxidative DNA damage compared to the disease + treatment groups, as illustrated in Figure 5F. The results, as shown in Figure S3, indicate that prolonged exposure to Cu^2+^ for 16 weeks caused an increase in brain Cu^2+^ levels compared to the control group. Treatment with CUR suspension and CUR-NE demonstrated its chelation efficacy by significantly reducing Cu^2+^ concentrations in brain tissue, highlighting its potential therapeutic effect.

### 2.8. Histopathological Evaluation

In the brain histopathological assessment, the control group showed no signs of inflammation or cellular imbalance. In contrast, the diseased groups exhibited significant brain structural changes, including cellular swelling, vacuolation, myelin breakdown, and neuronal damage [28]. The Disease + CUR/CUR-NE groups displayed mild alterations in the myelin sheath, with fewer cellular abnormalities (Figure 5G–J). Importantly, both the CUR suspension and CUR-NE groups demonstrated healthier brain tissue compared to the Cu^2+^-treated rats [29]. The blue arrows indicate the absence of inflammation or cellular imbalance, representing healthy morphology in the control group. In contrast, the yellow arrows highlight signs of neuronal damage and structural alterations, reflecting pathological changes.

## 3. Materials and Method

Curcumin (99% purity) was purchased from Himedia Laboratory, India. Ginger oil was purchased from Sigma, Germany. Tween 80, PEG 400, and Transcutol P were purchased from SRL, Taloja. Ethanol and methanol were purchased from Merk, Mumbai. The 6-diamidino-2-phenylindole (DAPI) and FITC-dextran were purchased from Sigma-Aldrich (USA). The 3-(4,5-Dimethylthiazol-2-yl)-2,5-Diphenyltetrazolium bromide) (MTT), Dulbecco’s Modified Eagle Medium (DMEM), Tris-HCl buffer, fetal bovine serum, and penicillin-streptomycin (penstrep) antibiotics were obtained from Thermo Scientific™. Triple-distilled water, used in the formulation preparation, was sourced from the Milli-Q system (Millipore, Merck) and was in-house. Biochemical assay kits were purchased from Bioassay Technology Laboratory.

### 3.1. Pre-Formulation Studies

#### 3.1.1. Solubility Analysis of Curcumin

The solubility of CUR was evaluated in various oils (olive, clove, and ginger oil), surfactants (Tween 40, 60, 80, and Span series), and cosurfactants (PEG 400, ethanol, Cremophor EL, Transcutol P, and soy lecithin). CUR was added to 1.5 mL of each selected oil, surfactant, and cosurfactant mixture in microcentrifuge tubes. The tubes were vortexed using a Spinix vortex mixer to ensure complete dispersion. To achieve equilibrium, the tubes were incubated at 37 ± 1.0 °C in a shaking water bath for 72 h [30]. After completion of 72 h, the samples were diluted in methanol and quantified using UV spectroscopy at 425 nm [31].

#### 3.1.2. Design of Pseudo-Ternary Phase Diagram

The pseudo-ternary phase diagram assists in the understanding of the spontaneous emulsification process. The pseudo-ternary point diagram demonstrated clear, turbid, and biphasic states, which were coded with colors. Ginger oil, Tween 80 as a surfactant, Transcutol P, and ethanol as a cosurfactant was used in NEs components after the assessments of different oils, surfactants, and cosurfactants on the basis of miscibility and solubility analysis. The pseudo-ternary phase diagrams were designed using oil, S_mix_, and distilled water and were built using the aqueous titration method. For the selected Tween 80 and DGME, ethanol was mixed in different ratios of 1:1, 1:2, and 2:1. Each oil: S_mix_ ratio was slowly titrated with a water phase, and visual inspection of the samples was done for indications of separation of the phases [32].

### 3.2. Preparation of Curcumin-Loaded Nanoemulsion (CUR-NE)

CUR-loaded nanocarriers were synthesized using a spontaneous emulsification technique, which involved the gradual addition of an aqueous phase into a pre-mixed oil and surfactant blend under controlled conditions with gentle stirring to form stable O/W nanoemulsions. The emulsification process is governed by factors such as phase transition regions, interfacial tension, viscosity, structural properties, and the concentration of surfactants. The preparation method was executed in three stages: initially, an organic solution was prepared by dissolving 4 mg of CUR in 5% ginger oil. Subsequently, a surfactant system comprising hydrophilic surfactant (Tween 80) and cosurfactant (ethanol) was formulated. Finally, the aqueous phase was introduced dropwise into the CUR-containing oily phase while stirring continuously at room temperature to achieve uniform emulsification. This method yielded CUR-encapsulated nanocarriers with desirable characteristics for potential applications [33].

#### Thermodynamic Stability Analysis

A thermodynamic stability study was accomplished for the various batches of nanoemulsion manufactured by varying the concentration of stabilizer and water chosen from the pseudo-ternary phase diagrams at different stress conditions [34]. The stability of the samples was analyzed through three different tests: the heating–cooling cycle, centrifugation, and freeze–thaw cycle. The heating–cooling cycle test involved subjecting the formulations to six temperature series ranging from 4 °C to 45 °C, with each temperature maintained for at least 48 h. Following this, a centrifugation test was conducted on the formulations to evaluate their stability and identify those that remained stable under these conditions. To evaluate the stability of the samples, batches were first centrifuged at 3500 rpm for 30 min. Samples that showed no signs of instability after centrifugation were then subjected to a freeze–thaw cycle. This test involved subjecting the samples to repeated freezing and thawing to check for any phase separation or instability under temperature changes. Three consecutive freeze–thaw cycles were conducted at temperatures ranging from −20 °C to room temperature over a 48 h period for the storage of samples. Only those samples that remained clear and did not exhibit any phase separation were selected for further analysis.

### 3.3. Characterization of CUR-NE

#### 3.3.1. Droplet Size and Surface Charge Analysis

The droplet diameter, PDI, and zeta potential were assessed using Zetasizer (Malvern Instruments Ltd., Worcester, UK). A total of 200 µL test samples of nanocarriers were mixed with 2 mL triple-distilled water, and the measurement was taken in triplicate by using normal disposable cuvettes for size and disposable folded capillary cuvette for zeta potential at 25 °C temperature [34].

#### 3.3.2. Drug Contents

The nanoformulation was subjected to centrifugation at 1200 rpm, and the resulting pellet was resuspended in methanol. The drug content was determined using HPLC (Agilent 1100 series, (Agilent Technologies, Memphis, TN, USA) at 425 nm. The percentage of drug content in the CUR-NE was calculated using the following formula:

$$\%\;\mathrm{Drug}\;\mathrm{contents}=\frac{\mathrm{Amt}.\;\mathrm{of}\;\mathrm{CUR}\;\mathrm{measured}\;\mathrm{by}\;\mathrm{Spectrophotomer}}{\mathrm{Total}\;\mathrm{amount}\;\mathrm{of}\;\mathrm{CUR}\;\mathrm{added}}*100$$

#### 3.3.3. FTIR Analysis

The compatibility of excipients and the purity of the formulation were assessed using FT-infrared spectroscopy (Bruker Alpha-P FTIR, Bruker, Boston, MA, USA). A sample consisting of the drug and excipients was placed on the sample holder, and the clean probe was positioned accordingly. The analysis was performed over a wavelength range of 450 to 4000 cm^−1^ to evaluate the interactions and determine the purity of the formulation [35]. The obtained data were evaluated on OPUS software version 9.0 and plotted.

#### 3.3.4. Morphological Characterization of Prepared Nanocarrier

Scanning electron microscopy (SEM, JEOL JSM-IT 200, Tokyo, Japan) was employed to assess the droplet size and surface morphology of the nanoemulsion. A 15 µL aliquot of the sample was placed on a stub covered with carbon tape and allowed to dry in a vacuum desiccator for 3 days. Prior to imaging, the sample was gold-coated for 2 min to enhance conductivity. The prepared sample was then mounted onto the sample holder, and its morphology was examined under low voltage using the SEM [36].

#### 3.3.5. Stability Studies

The accelerated stability of the CUR-NE was assessed by storing the formulation at temperatures of 45 °C, 25 °C, and 4 °C for one month. Particle size, PDI, and zeta potential were measured using dynamic light scattering (DLS) at regular time intervals to track any changes in the formulation’s stability over time under different storage conditions [37].

#### 3.3.6. In Vitro Drug Release Studies

The release profiles of CUR and CUR-NE were assessed using a dialysis bag with a molecular weight cutoff of 12,000 Dalton. The study was conducted in three different pH conditions (pH 1.2, 6.8, and 7.4) to simulate physiological environments. The dialysis bag was pre-soaked overnight in the release medium to hydrate the membrane. A 2 mL aliquot of CUR and CUR-NE (equivalent to 4 mg) was placed in the dialysis bag and submerged in 100 mL of release medium, which was continuously stirred at 100 rpm and maintained at 37 ± 0.5 °C. At specified time intervals (0.5, 1, 2, 4, 8, 12, and 24 h), 2 mL samples were withdrawn from the release medium and replaced with fresh medium to ensure sink conditions. The drug concentration was measured using UV–visible spectrophotometry at 425 nm [38,39].

#### 3.3.7. Permeability Studies

The intestinal permeability of free drug and nanoformulation was evaluated by utilizing the non-everted intestinal (rats) sac technique. The intestine was taken from sacrificed SD rat of the control group from an animal laboratory. The small intestine was freed from intestinal content by passing the oxygenated cold normal saline solution with the blunt syringe. Cleaned intestinal segments were cut into 8 cm long pieces and placed into a Krebs Ringers buffer (7.4 pH) solution with oxygenation. A blunt needle was used to inject each sac with an equivalent concentration of CUR suspension and CUR-NE, and both sides of the intestine were then securely tied with silk thread. This non-everted intestine was placed in beakers having 100 mL of Krebs Ringer buffer solution (7.4 pH) on a magnetic stirrer with 100 rpm at 37 °C temperature equipped with laboratory aerators. An amount of 5 mL of aliquot was taken at programmed time intervals and replaced with the same volume of KRB to keep the sink state [40]. The samples were investigated by using Agilent 1100 series HPLC, (Memphis, TN, USA) techniques at 425 nm at room temperature. The cumulative amount permeated per unit area (µg/cm^2^) of intestinal sacs was assessed.

### 3.4. Cell Line Protocol

The SH-SY5Y cell line was obtained from institutional cell line repository. Cells were seeded in the T25 flask under 5% CO_2_ incubator. The media composition consists of equal volumes of Minimum Essential Medium (MEM) and Dulbecco’s Modified Eagle Medium (DMEM) supplemented with 0.1% penicillin–streptomycin solution.

#### 3.4.1. Cell Viability Analysis

Cells were cultured in a 96-well plate and allowed to adhere for 12 h. After cell adhesion, the complete medium was substituted with incomplete medium, and treatments were carried out in two distinct experimental setups. In the first setup, cells were exposed to Cu^2+^ at varying concentrations of 1, 10, 20, 40, and 50 µM. In the second setup, cells were subjected to different treatment combinations, including Cu^2+^ at 50 µM alone, Cu^2+^ at 50 µM with CUR suspension at 2.5 µM, Cu^2+^ at 50 µM with CUR suspension at 5 µM, Cu^2+^ at 50 µM with CUR-NE at 2.5 µM, and Cu^2+^ at 50 µM with CUR-NE at 5 µM. After 24 h, the media were replaced with MTT solution, and cell viability was assessed using a multiplate reader (Synergy H1, BioTek, Woburn, MA, USA). Wells treated with 0.1% DMSO served as the 100% viability control, while Cu^2+^ (50 µM) acted as the positive control. The percentage of cell viability was calculated using the following formula. All the readings were calculated in triplicates. Data were analyzed using one-way ANOVA with Dunnett’s multiple comparison. All treatment groups were statistically compared to the control group to assess differences in the measured outcomes (denoted with *). The CUR vs. CUR-NE groups were statistically analyzed using a one-way ANOVA followed by the Sidak multiple comparisons test. Cell viability was defined according to the following equation:

*Cell viability* = *[Absorbance of treatment group/Absorbance of control group]* × 100

#### 3.4.2. Cellular Uptake Analysis

The SH-SY5Y cells were seeded in 12-well plates and allowed to adhere for 12 h. Following the attachment period, the cells were treated with FITC-labeled nanoemulsion (NE) for 3, 6, and 24 h in a CO_2_ incubator. After each treatment interval, the cells were stained with DAPI for 5 min, followed by removal of the DAPI solution and washing with fresh PBS. The coverslips were then mounted onto glass slides with 80% glycerol and sealed with clear gel polish. The slides were examined using confocal microscopy (Leica Microsystems DMI8, Wetzlar, Germany), with images captured in both the green and blue channels to assess the cellular uptake and distribution of the NE.

### 3.5. Animal Study Protocol

Sprague Dawley rats (SD) with 180 to 250 g weight range were selected. The animals were acclimatized for 7 days and then were sorted via further randomization for treatment. The pharmacokinetic and pharmacodynamic study (protocol no. NIPER/RBL/IAEC 192 and 8 March 2024) was approved by the Institutional Animal Ethics Committee (IAEC) at NIPER-Raebareli.

#### 3.5.1. Pharmacokinetic Analysis

Following an overnight fast, the rats were given an oral dose of CUR suspension at 80 mg/kg (prepared in 0.5% sodium carboxymethyl cellulose) and an equivalent dose of CUR-NE (at equivalent concentration). The blood collection was carried via retroorbital route under slight anesthesia in the following intervals: 0.25, 0.5, 1, 2, 4, 8, 12, and 24 h in EDTA tubes. After 24 h, the rats were euthanized, and tissues like brain, kidneys, liver, and lungs were isolated. These tissues were rinsed with PBS (pH 7.4), dried, weighed, and homogenized in PBS using a tissue homogenizer. The resulting homogenates were stored at −80 °C till evaluation. The samples were analyzed with HPLC analysis (emodin was used as internal standard at 510 nm and CUR at 425 nm. The mobile phase was composed of 0.05 M sodium acetate buffer: Acetonitrile at 60:40 ratio at 1 mL per minute. The isolated homogenate samples were centrifuged with 500 µL acetonitrile at 12,000 rpm for 5 min at 4 °C. The separated organic phase was evaporated to dryness and reconstituted with acetonitrile and evaluated on HPLC instrument. The obtained data were analyzed with the PKSolver software version 10. All the readings were calculated in (*n* = 6). Data were analyzed using one-way ANOVA with Dunnett’s multiple comparison. All treatment groups were statistically compared with the control group to assess differences in the measured outcomes (denoted with *). The CUR vs. CUR-NE groups were statistically analyzed using a one-way ANOVA followed by the Sidak multiple comparisons test (denoted with #). The drug targeting index was defined according to the following equation:

$$Drug\;targeting\;Index=\frac{\left(\;AUC1\right)B1/\left(AUC1\right)p1}{\left(AUC2\right)B2/\left(AUC2\right)p2}$$

B1 represents the AUC of CUR-NE in brain;
B2 represents the AUC of CUR-suspension in brain;
P1 represents the AUC of CUR-NE in plasma;
P2 represents the AUC of CUR suspension in plasma.

#### 3.5.2. Pharmacodynamic Study

The animals were randomly assigned to four groups. In Group I (control), SD rats received daily intraperitoneal (IP) injections of 0.9% NaCl. Group II was treated with Cu^2+^ (20 mg/kg) daily for 16 weeks in drinking water. In Group III, rats were given Cu^2+^ (20 mg/kg) daily for 16 weeks, followed by 14 days of oral CUR treatment at 80 mg/kg. Group IV received Cu^2+^ (20 mg/kg) daily for 16 weeks, followed by 14 days of oral CUR-NE treatment at 80 mg/kg. Thus, the Cu^2+^ treatment lasted 16 weeks, after which the CUR and CUR-NE treatments were administered for 14 days. All the readings were calculated in (*n* = 6). Data were analyzed using one-way ANOVA with Dunnett’s multiple comparison. All treatment groups were statistically compared to the control group to assess differences in the measured outcomes (denoted with *). The CUR vs. CUR-NE groups were statistically analyzed using a one-way ANOVA followed by the Sidak multiple comparisons test (denoted with #).

**(i).**  **Morris water maze test**

The Morris water maze (MWM) test was employed to assess learning and memory performance after exposure to Cu^2+^ and subsequent treatments. Five rats from each group participated in a four-day training period, during which they explored various quadrants to locate a hidden platform submerged in the water. The time taken to reach the platform was recorded during each trial. On the fifth day, a probe trial was conducted to evaluate memory retention, measuring the time taken to identify the target quadrant and the duration spent within it. Following the behavioral tests, the rats were sacrificed, and tissue samples were collected for further analysis [41].

**(ii).**  **Nobel Object Recognition Test**

The Novel Object Recognition Test (NORT) was conducted to evaluate the effects of Cu^2+^ toxicity on memory function. The setup included a black open-top box measuring 65 cm × 65 cm × 45 cm, with a high-definition camera (Lenovo 300 FHD Webcam) placed above to record the rats’ behavior. The test was divided into three stages: habituation, familiarization, and recognition. During the habituation stage, rats were allowed to explore the empty box freely. In the familiarization stage, they were introduced to two identical objects placed within the box. Finally, in the recognition phase, one of the familiar objects was replaced with a novel object, and the time spent exploring each object was recorded [42].

### 3.6. Assessment of Oxidative Stress Biomarkers in the Brain

**(i).**  **Quantification of Superoxide Dismutase Quantity**

The brain homogenate was prepared by suspending the tissue in Tris-HCl buffer, followed by centrifugation (sigma Z-16pk, Osterode, Germany) to collect the supernatant. For the assay, a reaction mixture was prepared by combining 0.2 mL of the brain supernatant with 0.8 mL of distilled water and 0.2 mL of NADH. After incubation, the reaction was terminated by adding acetic acid. The mixture was allowed to stand for 10 min, and the absorbance was measured at 560 nm using a spectrophotometer to assess the intensity of the reaction.

**(ii).**  **Quantification of Catalase Activity**

CAT activity in brain tissue was measured by combining the homogenate with 1 mL of substrate solution. The mixture was allowed to incubate for 2 min, after which the reaction was terminated with 1 mL of ammonium molybdate, resulting in the formation of a yellow-colored complex. The intensity of the color was then quantified by measuring the absorbance using a spectrophotometer.

**(iii).**  **Quantification of MDA**

To quantify thiobarbituric acid-reactive substances (TBARSs) in brain tissue, 100 μL of brain homogenate was combined with 250 μL of 20% acetic acid, 250 μL of thiobarbituric acid, and 8% sodium dodecyl sulfate, followed by the addition of distilled water to reach the desired volume in a test tube. The mixture was incubated at 90 °C for 1 h and then rapidly cooled under running tap water. After cooling, the samples were centrifuged, and the absorbance was measured at 532 nm using a spectrophotometer. The results were compared to a standard curve for quantification.

**(iv).**  **Quantification of Nitric oxide Levels**

Brain nitric oxide levels were measured using the Griess reagent. To perform the test, 100 μL of the reagent was combined with equivalent volume of brain homogenate. After incubation, the resulting reaction product was analyzed at 540 nm using a spectrophotometer. A standard curve was created using sodium nitrite, and the nitrite levels in the brain samples were calculated and reported in micromoles per milligram of protein.

**(v).**  **Estimation of ROS**

The DCFDA assay was employed to assess oxidative stress levels in brain tissue. For the assay, brain homogenate was mixed with DCFDA solution and 980 µL of buffer (pH 7.4). The mixture was incubated in the dark for 20 min to prevent light interference. Fluorescence measurements were obtained using a multimode plate reader, with excitation at 483 nm and emission at 530 nm. The oxidative stress levels were quantified as fluorescence units (FU) per milligram of protein.

**(vi).**  **Quantification of Neuroinflammatory Marker (8-OHdG)**

To assess the levels of 8-hydroxy-2′-deoxyguanosine (8-OHdG) in brain tissue, the homogenate was combined with a commercially available pre-mixed solution and incubated for 20 min. Following incubation, 200 μL of Folin–Ciocalteu reagent was introduced to the mixture, and the incubation continued. The absorbance of the resulting reaction was measured using a spectrophotometer, and the concentration of 8-OHdG was calculated by referencing a standard curve.

**(vii).**  **Assessment of cerebral Cu^2+^ content**

Brain Cu^2+^ levels were quantified using inductively coupled plasma mass spectrometry (ICP-MS). In summary, brain tissue samples were subjected to a standard acid digestion process. The resulting digested solutions were analysed for Cu^2+^ concentration using a PerkinElmer emission spectrometer.

All the readings were calculated in (*n* = 6). Data were analyzed using one-way ANOVA with Dunnett’s multiple comparison. All treatment groups were statistically compared to the control group to assess differences in the measured outcomes (denoted with *). The CUR vs. CUR-NE groups were statistically analyzed using a one-way ANOVA followed by the Sidak multiple comparisons test (denoted with #).

### 3.7. Histopathological Evaluations

After the behavioural studies, the animals were sacrificed, and their brains were carefully extracted. The tissues were then placed in a fixation solution until they became firm. Following fixation, the brain tissue was embedded in paraffin wax and allowed to solidify in a mold. Once solidified, the tissue was sliced into 5 mm thick sections. These sections underwent a series of alcohol washes before being stained with hematoxylin and eosin. Finally, the stained slides were examined under a microscope for analysis [2,43].

## 4. Conclusions

In this study, we highlight the significant potential of CUR-NE in overcoming the challenges associated with CUR’s therapeutic application in neurodegenerative diseases. Our developed nanoformulation effectively enhances CUR’s pharmacokinetic profile and improves its systemic bioavailability, which is crucial for targeting neurological conditions. By amplifying CUR ’s anti-inflammatory, antioxidant, and metal chelation properties, this nanoemulsion offers a multifaceted approach to addressing metal-induced neurodegeneration. The inclusion of synergistic agents like ginger oil further strengthens the therapeutic impact, potentially improving outcomes for diseases linked to oxidative stress and metal toxicity. Our findings, including improvements in cognitive function and memory in rat models, suggest the broader applicability of developed formulations in treating Cu^2+^ induced neurodegenerative disorders. We recognize the need for further investigation, particularly regarding long-term safety, optimal dosing, and efficacy in clinical studies. Overall, this work emphasizes the growing role of nanotechnology in enhancing the effectiveness of natural compounds, offering new avenues for the treatment of complex neurological diseases.

## Figures and Tables

**Table 1:** Thermodynamic stability testing of NE region obtained from pseudo-ternary phase diagram using Tween 80 and ethanol (1:1) (+ indicates stability, whereas − indicates instability).

Batch No.	% *v*/*v* of Solvent			Heating and Cooling Cycle	Centrifugation	Freeze–Thaw Cycle
	Oil	Smix	Water			
A1	5	25	70	+	−	−
A2	5	30	65	+	+	−
A3	5	35	60	+	+	+
A4	5	40	55	+	+	+
A5	5	45	50	+	+	+
A6	5	50	45	+	+	+
A7	5	55	40	+	−	−
A8	5	60	35	−	−	−
A9	5	65	30	+	+	+
A10	5	70	25	−	+	−
A11	10	40	50	+	−	−
A12	10	45	45	+	+	+
A13	10	50	40	+	+	+
A14	10	55	35	−	−	−
A15	10	60	30	−	+	+
A16	10	65	25	+	+	−
A17	15	55	30	+	−	−
A18	15	60	25	+	+	+
A19	15	65	20	+	−	−
A20	15	70	15	+	+	+

**Table 2:** Stability data.

Sampling Time	Size (nm) ± SD	PDI ± SD	ZP (mV)
			±SD
At 4 °C			
Initial	76.42 ± 0.09	0.192 ± 0.79	−20.5 ± 0.75
After 10 days	76.42 ± 0.09	0.245 ± 0.67	−20.2 ± 0.43
After 20 days	76.12 ± 0.45	0.217 ± 1.56	−19.5 ± 0.48
After 30 days	75.56 ± 0.79	0.282 ± 0.71	−18.1 ± 1.18
At Room Temperature			
Initial	76.42 ± 0.11	0.266 ± 0.43	−20.4 ± 0.47
After 10 days	76.50 ± 0.74	0.200 ± 0.76	−20.4 ± 0.51
After 20 days	77.19 ± 0.81	0.268 ± 0.44	−20.3 ± 0.93
After 30 days	76.98 ± 1.23	0.289 ± 0.21	−20.0 ± 0.71
At 45 °C			
Initial	76.42 ± 0.29	0.200 ± 0.09	−20.7 ± 0.86
After 10 days	77.30 ± 0.76	0.219 ± 0.37	−20.3 ± 0.73
After 20 days	77.45 ± 0.83	0.242 ± 0.80	−19.4 ± 0.69
After 30 days	78.41 ± 1.44	0.282 ± 0.91	−18.19 ± 0.85

**Table 3:** Pharmacokinetic parameters and CUR quantification in plasma and brain.

Parameter	Units	Plasma		Brain	
		CUR Suspension	CUR-NE	CUR Suspension	CUR-NE
**t1/2**	**1/h**	5.63	8.71	6.5	9.99
**Tmax**	**h**	8	4	9	4
**Cmax**	**h**	1507.33	1207.35	373.92	1229.64
**AUC 0-t**	**ng/mL·h**	15,865.64	12,055.48	2719.98	16,330.34
**MRT 0-inf_obs**	**h**	15.56	8.73	8.99	11.35

## Data Availability

The data that support the findings of this study are available from the corresponding author upon reasonable request.

## Supplementary Materials

The following supporting information can be downloaded at https://www.mdpi.com/article/10.3390/toxics13020108/s1, Figure S1: Solubility analysis: (A) Solubility in co-surfactants (B) Solubility in surfactants. All data were calculated in triplicate, Figure S2: Ternary phase diagram (A) Tween 80: DEME in 1:1, 1:2 and 2:1 ratio, (B) Tween 80: ethanol in 1:1, 1:2 and 2:1 ratio, (C) PEG400: ethanol in 1:1, 1:2 and 2:1 ratio, Figure S3: Evaluation of cerebral copper content in different group (N = 3). All the readings were calculated in triplicates. Data was analyzed using one-way ANOVA with Dunnett’s multiple comparison. All treatment groups were statistically compared to the control group to assess differences in the measured outcomes (denoted with *). The CUR vs. CUR-NE groups were statistically analyzed using a one-way ANOVA followed by the Sidak multiple comparisons test.
