# Supplementary material for: Copper-Induced Neurodegenerative Disorders and Therapeutic Potential of Curcumin-Loaded Nanoemulsion
Source: Toxics. 2025 Jan 29;13(2):108. doi: 10.3390/toxics13020108 (PMC11862003; doi:10.3390/toxics13020108)
Supplement: Supplementary file 1 [file toxics-13-00108-s001.zip › toxics-3428834-supplementary.pdf]

Therapeutic Potential of Curcumin-Loaded Nanoemulsion in Counteracting Copper Toxicity in Neurodegenerative Disorders

Figure S1:

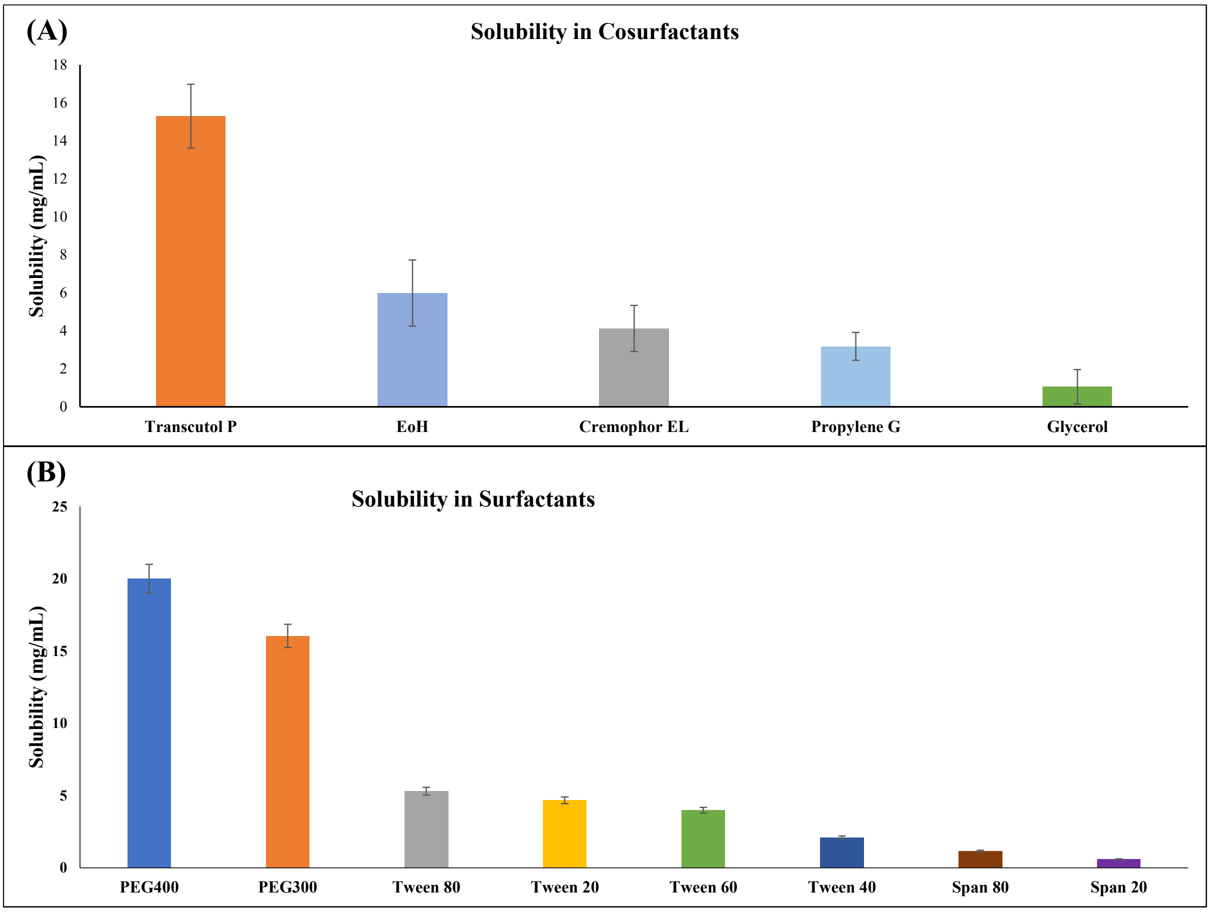

Figure S1: Solubility analysis: A) Solubility in co-surfactants B) Solubility in surfactants. All data were calculated in triplicates.

Figure S2:

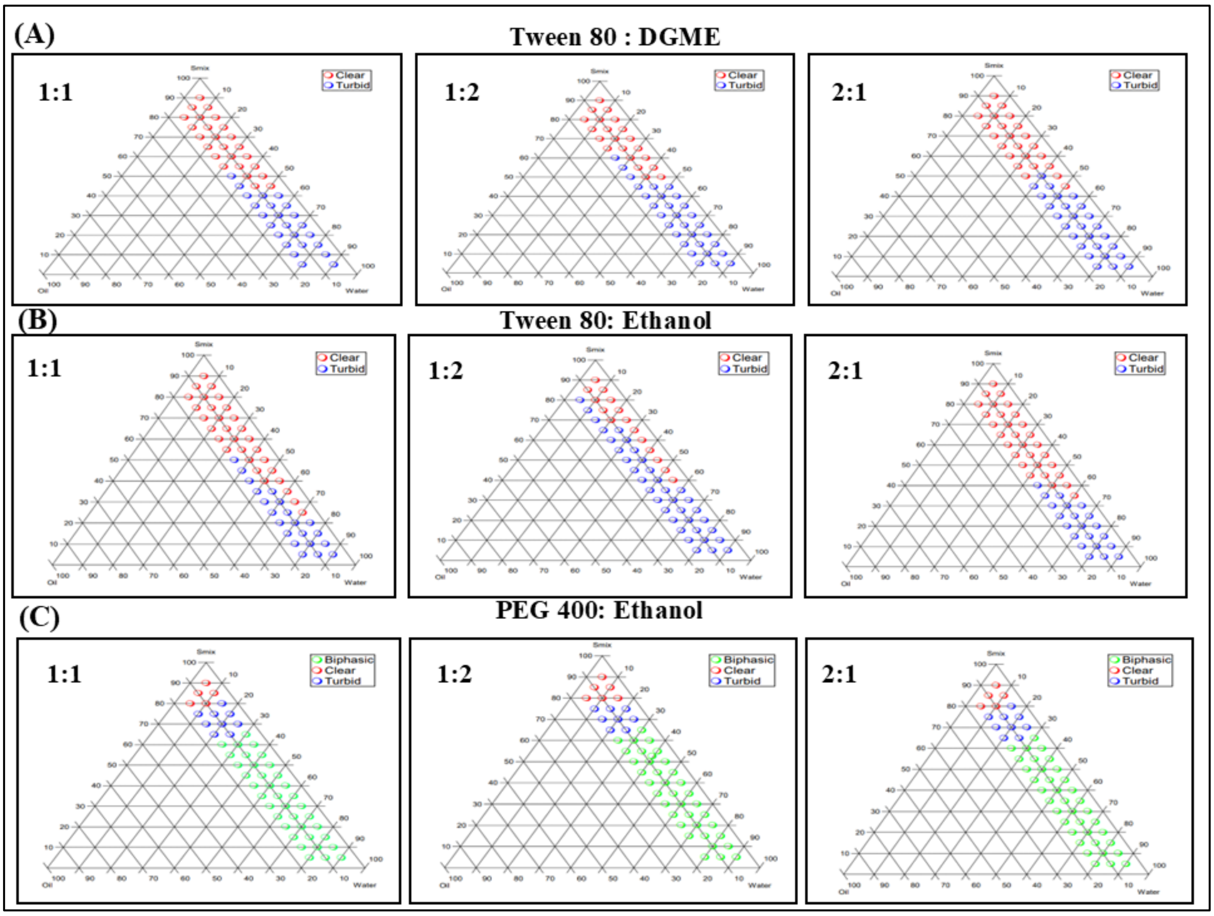

Figure S2: Ternary phase diagram A) Tween 80: DEME in 1:1, 1:2 and 2: 1 ratio, B) Tween 80: ethanol in 1:1, 1:2 and 2: 1 ratio, C) PEG400: ethanol in 1:1, 1:2 and 2: 1 ratio.

Figure S3:

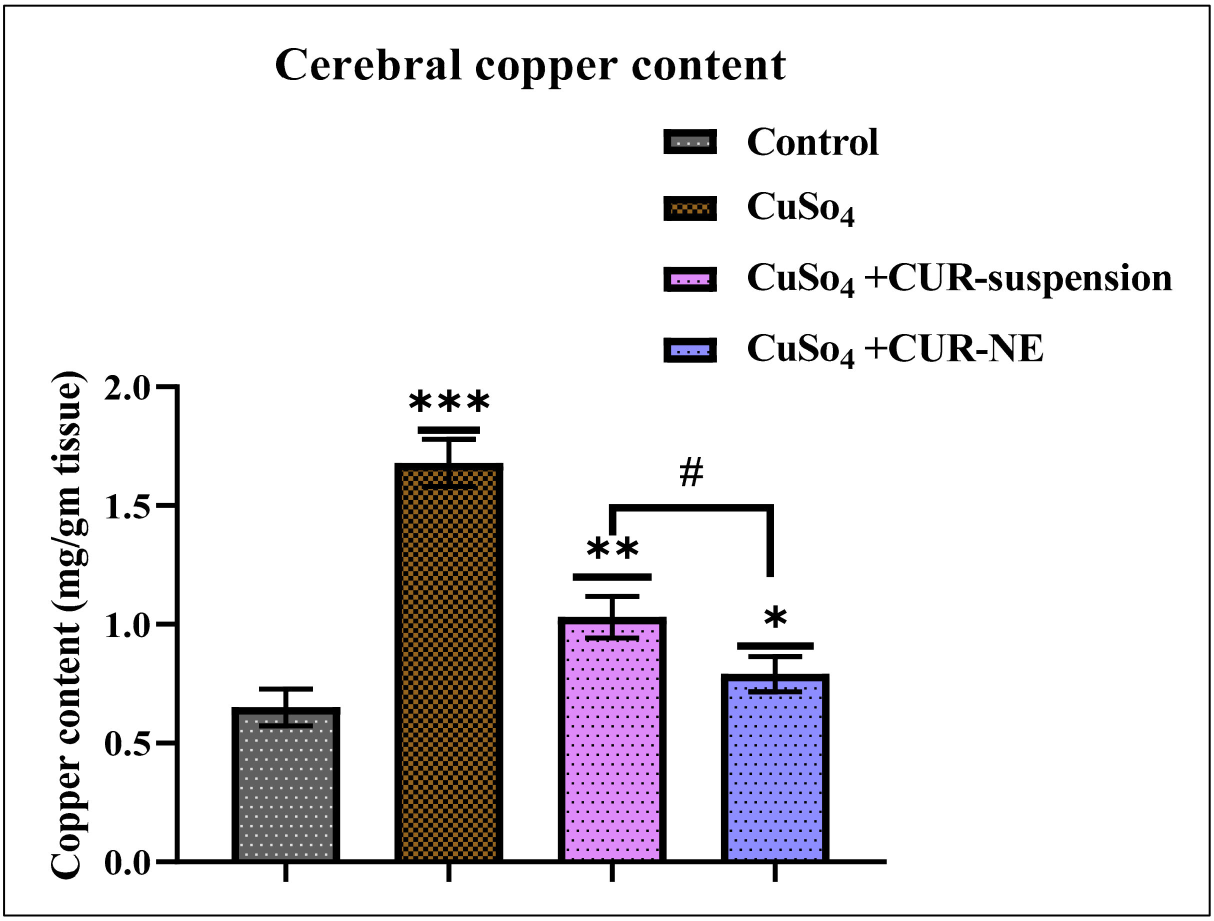

Figure S3: Evaluation of cerebral copper content in different group (N=3). All the readings were calculated in triplicates. Data was analyzed using one-way ANOVA with Dunnett's multiple comparison. All treatment groups were statistically compared to the control group to assess differences in the measured outcomes (denoted with *). The CUR vs. CUR-NE groups were statistically analyzed using a one-way ANOVA followed by the Sidak multiple comparisons test.
